# Rapid extended-spectrum beta-lactamase-confirmation by using a machine learning model directly on routine automated susceptibility testing results

**DOI:** 10.3389/fmicb.2025.1582703

**Published:** 2025-04-30

**Authors:** Y. El Ghouch, M. C. Schut, K. C. E. Sigaloff, W. Altorf-Van Der Kuil, J. M. Prins, R. P. Schade, the ISIS-AR study group, J.W.T. Cohen Stuart

**Affiliations:** Noordwest Ziekenhuisgroep, Department of Medical Microbiology, Alkmaar; Meander Medical Center, Department of Medical Microbiology, Amersfoort; Amsterdam UMC, University of Amsterdam, Department of Medical Microbiology and Infection Prevention, Amsterdam Infection and Immunity Institute, Amsterdam; Atalmedial, Department of Medical Microbiology, Amsterdam; OLVG Lab BV, Department of Medical Microbiology, Amsterdam; Public Health Service, Public Health Laboratory, Amsterdam; Gelre Hospitals, Department of Medical Microbiology and Infection prevention, Apeldoorn; Centre for Infectious Disease Control (CIb), National Institute for Public Health and the Environment (RIVM), Bilthoven, Netherlands; Microvida Amphia, Laboratory for Microbiology and Infection Control, Breda; IJsselland hospital, Department of Medical Microbiology, Capelle a/d Ijssel; Reinier de Graaf Group, Department of Medical Microbiology, Delft; Deventer Hospital, Department of Medical Microbiology, Deventer; Slingeland Hospital, Department of Medical Microbiology, Doetinchem; Albert Schweitzer Hospital, Department of Medical Microbiology, Dordrecht; Gelderse Vallei Hospital, Department of Medical Microbiology, Ede; Admiraal De Ruyter Hospital, Department of Medical Microbiology, Goes; Groene Hart Hospital, Department of Medical Microbiology and Infection Prevention, Gouda; Haaglanden MC, Department of Medical Microbiology, ‘s-Gravenhage; Haga Hospital, Department of Medical Microbiology, ’s-Gravenhage; Certe, Medical Microbiology Groningen|Drenthe, Groningen; University of Groningen, University Medical Center, Department of Medical Microbiology, Groningen; University of Groningen, University Medical Center, Department of Medical Microbiology, Groningen; Regional Public Health Laboratory Haarlem, Haarlem; St Jansdal Hospital, Department of Medical Microbiology, Harderwijk; St Jansdal Hospital, Department of Medical Microbiology, Harderwijk; Jeroen Bosch Hospital, Department of Medical Microbiology and Infection Control, ’s-Hertogenbosch; CBSL, Tergooi MC, Department of Medical Microbiology, Hilversum; CBSL, Tergooi MC, Department of Medical Microbiology, Hilversum; Comicro, Department of Medical Microbiology, Hoorn; Certe, Medical Microbiology Friesland|NOP, Leeuwarden; Leiden University Medical Center, Department of Medical Microbiology, Leiden; Eurofins Clinical Diagnostics, Department of Medical Microbiology, Leiden-Leiderdorp; Maastricht University Medical Centre, Department of Medical Microbiology, Infectious Diseases and Infection Prevention, Maastricht; St Antonius Hospital, Department of Medical Microbiology and Immunology, Nieuwegein; Canisius Wilhelmina Hospital, Department of Medical Microbiology and Infectious Diseases, Nijmegen; Radboud University Medical Center, Department of Medical Microbiology, Nijmegen; Laurentius Hospital, Roermond; Bravis Hospital, Department of Medical Microbiology, Roosendaal; Erasmus University Medical Center, Department of Medical Microbiology and Infectious Diseases, Rotterdam; Franciscus Gasthuis and Vlietland, Department of Medical Microbiology and Infection Control, Rotterdam; Ikazia Hospital, Department of Medical Microbiology, Rotterdam; Maasstad Hospital, Department of Medical Microbiology, Rotterdam; Star-SHL, Rotterdam; Zuyderland Medical Centre, Department of Medical Microbiology and Infection Control, Sittard-Geleen; Zuyderland Medical Centre, Department of Medical Microbiology and Infection Control, Sittard-Geleen; Microvida ZorgSaam, Department of Medical Microbiology, Terneuzen; Diakonessenhuis, Department of Medical Microbiology and Immunology, Utrecht; Saltro Diagnostic Centre, Department of Medical Microbiology, Utrecht; University Medical Center Utrecht, Department of Medical Microbiology, Utrecht; Eurofins-PAMM, Department of Medical Microbiology, Veldhoven; Rijnstate Hospital, Laboratory for Medical Microbiology and Immunology, Velp; VieCuri Medical Center, Department of Medical Microbiology, Venlo; Isala Hospital, Laboratory of Medical Microbiology and Infectious Diseases, Zwolle; ^1^Department of Medical Microbiology and Infection Prevention, Amsterdam UMC, Amsterdam, Netherlands; ^2^Department of Internal Medicine, Amsterdam UMC, Amsterdam, Netherlands; ^3^Department of Laboratory Medicine, Amsterdam UMC, Amsterdam, Netherlands; ^4^Centre for Infectious Disease Control (CIb), National Institute for Public Health and the Environment (RIVM), Bilthoven, Netherlands

**Keywords:** ESBL, machine learning, antimicrobial resistance, bacteria, surveillance

## Abstract

**Objectives:**

Phenotypical Extended Spectrum β-Lactamase (ESBL)-production is commonly determined using the combination disk diffusion test or gradient test. This requires overnight incubation, prolonging time-to-detection and increasing duration of empirical treatment for patients with infections caused by gram-negative bacteria. To achieve instant confirmation without incubation, we developed a machine learning (ML)-model that predicts phenotypic ESBL-confirmation using Minimum Inhibitory Concentrations from routine automated antimicrobial susceptibility testing (AST)-results.

**Methods:**

Data from the Dutch national laboratory-based surveillance system ISIS-AR collected between 2013 and 2022 from 49 laboratories were used: 178,044 isolates of E. coli (141,576), K. pneumoniae (33,088), and P. mirabilis (3,380) that exhibited resistance to cefotaxime and/or ceftazidime, and had available results of phenotypical ESBL-confirmation testing. We evaluated Logistic Regression, Random Forest and XGBoost models and calculated SHAP-values (SHapley Additive exPlanations) to identify most contributing features. We externally validated models using 5,996 isolates collected in Amsterdam University Medical Centres’ between 2013 and 2022.

**Results:**

XGBoost achieved an AUROC (Area Under Receiver Operating Characteristics) of 0.97, a sensitivity of 0.89 and an accuracy of 0.93. The most contributing features were the antibiotics cefotaxime, cefoxitin and trimethoprim for *E. coli* and *K. pneumoniae*, and cefuroxime, imipenem and cefotaxime for *P. mirabilis*. External validation yielded AUROCs of 0.93 (E. coli), 0.89 (K. pneumoniae) and 0.93 (P. mirabilis).

**Conclusion:**

ML-models for prediction of ESBL-production using routine AST-system data achieved high performances. Implementing these models in laboratory practice could shorten time-to-detection. Once deployed, this approach could facilitate widespread screening for phenotypic ESBL-production.

## Introduction

Extended-spectrum β-lactamase (ESBL) producing gram-negative bacteria are a global health burden, and the predominant cause of third-generation cephalosporin resistance in Europe (Livermore et al., 2007). The genes that encode for ESBL-enzymes are mostly located on behalf of the ISIS-AR study group. plasmids, which facilitate transfer between bacterial strains and dissemination between patients. For these reasons, early and reliable detection of ESBL-producing bacteria is important. A wide variety of gram-negative bacteria can harbor ESBL-enzymes. The most predominant ESBL-producing species are *E. coli*, *K. pneumonia*, and *P. mirabilis* (Bradford, 2001; Castanheira et al., 2021). In these species, guidelines recommend that a clinical isolate exhibiting resistance to third-generation cephalosporins during initial testing and reporting, should be screened for ESBL-production (Clinical and Laboratory Standards Institutes (CLSI), 2024; EUCAST, 2017; NVMM, 2021). A minimal inhibitory concentration (MIC) that is larger than 1 μg/ml for cefotaxime and/or ceftazidime are advised as the threshold for ESBL-confirmation testing (EUCAST, 2017; NVMM, 2021). Phenotypic ESBL-confirmation is performed using either the combination disk diffusion test or the gradient test (NVMM, 2021). In these tests, susceptibility for certain cephalosporins is tested in the presence and absence of clavulanic-acid, a potential ESBL-inhibitor.

Currently, the process of ESBL-screening and confirmation has several limitations. Firstly, for practical reasons, screening is performed based on results of only three selected cephalosporins. However, for many other β-lactams there is a correlation between increased MICs and ESBL-presence (Bradford, 2001; Castanheira et al., 2021). In addition, ESBL-positive isolates often harbor co-resistance to other antibiotics such as aminoglycosides and quinolones, but this potentially relevant information is not incorporated into the process. More importantly, the confirmation of ESBL production requires overnight incubation, which significantly delays time-to-detection. It is customary to treat a patient with suspected ESBL-infection by initiating broad-spectrum empirical therapy covering ESBL while awaiting definitive culture results, which currently takes at least one additional day.

The potential information loss and prolonged time-to-detection could be addressed with machine learning (ML). Recent years saw the introduction of several promising ML-model applications for clinical microbiology (Abu-Aqil et al., 2023; Lee et al., 2021; Lee et al., 2023), but to date no study has reported a model to predict the outcome of the ESBL-confirmation test. The current time-to-detection can be drastically reduced with an ML-model (providing instant predictions) based on MIC-results of automated antimicrobial susceptibility testing (AST) systems that are readily available when the ESBL-confirmation test would be conducted.

The aim of this study was to reduce the time to ESBL-confirmation among patients with infections caused by gram-negative bacteria by means of a ML-model that can accurately predict the outcome of phenotypic ESBL-confirmation tests directly from MIC-results.

## Materials and methods

### Data source and population and study design

We performed a study using data that are being prospectively collected by the Dutch national laboratory-based surveillance system for antimicrobial resistance (ISIS-AR) (Altorf-van der Kuil et al., 2017). ISIS-AR is a combined initiative by the Dutch National Institute for Public Health and the Environment (RIVM) and the Dutch Society of Medical Microbiology (NVMM). In this system AST data are collected monthly from laboratories based in Netherlands.

We extracted data on three bacterial species: *E. coli*, *K. pneumonia*, and *P. mirabilis*. The included isolates were sampled in the period 2013 until 2022 and collected from 49 different laboratories serving academic hospitals, general hospitals and non-hospital institutions. Species identification was performed by Maldi-Tof systems [Bruker MALDI biotyper^®^ (Bruker) or Vitek MS^®^ (BioMerieux)]. Inclusion criteria were availability of results from automated AST and phenotypic ESBL-confirmation.

Dutch national laboratory-based surveillance system for antimicrobial resistance requires that labs always should perform a confirmation test if the ESBL screenings criteria are met. We included isolates that we eligible for ESBL screening, i.e., exhibiting resistance to ceftazidime, cefotaxime or both as measured by the AST system (MIC > 1 μg/ml). Susceptibility testing results of the Vitek (BioMérieux, Durham NC) and Phoenix (BD Biosciences, Sparks, MD) systems were included, as these are the predominant systems used in Netherlands. Reference methods used for ESBL-confirmation were either combination disk diffusion test or commercial gradient tests (Etest^®^-BioMerieux, MIC Test Strip^®^-Liofilchem), as per the EUCAST and national directives (EUCAST, 2017; NVMM, 2021). We removed potential duplicates by including one isolate per patient per material per 6 months (Birgand et al., 2013). From these isolates, additional information about the site of origin of the isolate and the type of AST system was obtained.

The variables in the dataset represent MIC measured during susceptibility testing of the isolates. We included the MIC-values for antibiotics that are currently on the standard testing panels of the included AST-systems, namely: amoxicillin, amoxicillin-clavulanic acid, piperacillin-tazobactam, cefoxitin, cefuroxime, cefotaxime, ceftazidime, imipenem, meropenem, ciprofloxacin, colistin, co-trimoxazole, fosfomycin, gentamicin, tobramycin, nitrofurantoin, trimethoprim. An overview of the ranges of the measurable MIC-values on the testing panels can be found in the Supplementary Table 1.

### Model outcome

We developed a model to predict for an isolate whether the detected micro-organism produces ESBL or not.

### Model development

The dataset was divided into three subsets based on the three bacterial species because there is substantial variation in occurrences and a significant lower ESBL-prevalence for *P. mirabilis*. This allowed a detailed investigation into the characteristics and prevalence patterns within each subset, and development of the optimal model for each species. We did not include the AST-system (Vitek or Phoenix) as a separate feature as the objective was to develop a prediction model that can be used independent of the specific system.

Three different ML-algorithms were evaluated: Logistic Regression, Random Forest and XGBoost. Logistic Regression (Cox, 1958) is known for its simplicity, making it a strong baseline algorithm. Random Forest (Tin, 1995) and XGBoost (eXtreme Gradient Boosting) (Chen and Guestrin, 2016) are both ensemble learning algorithms that are known for their high predictive accuracy.

Each subset was subjected to each ML-algorithm, leading to the development of nine prediction models. To develop the models, we applied a stratified ten-fold cross-validation using an 80/20 division and averaging the results. The experiments were conducted in Python 3.9.7 with Pandas (1.3.4), Scikit-learn (0.24.2) and XGBoost (1.7.3).

### Performance measurement

To evaluate the performance of the developed models, we calculated multiple metrics. To assess how well the model discriminates between positive and negative, we calculated the AUROC (Area Under Receiver Operating Characteristics). For the calibration of the predictions, we plotted calibration curves and calculated the Brier score. A higher AUROC indicates better performance, while a lower Brier score indicates a better calibration. These metrics are independent of the decision threshold, s than 0.8 was defined as an ESBL-negative outcome. Using a higwhich is used to convert predicted probabilities to an ESBL-label. To define a model outcome as ESBL-positive, a decision threshold of 0.8 was used in this study: a probability of less threshold makes the model strict and, therefore, minimizes the rate of false positives. With this threshold, we calculated additional metrics: accuracy, f1-score and sensitivity. These are also indicators of how well the model discriminates between positives and negatives, but given a certain decision threshold. The definitions of the performance metrics are given in the Supplementary Table 2.

### Feature importance

We calculated SHAP-values (SHapley Additive exPlanations) (Lundberg et al., 2021) to assess the importance of each feature on the predictions. If the model performs well (i.e., classifies correctly), SHAP-values indicate which antibiotics are most relevant in the model predicting ESBL-production.

### External validation

To assess the robustness and quality of the models, they were validated on a separate data-cohort. For this external validation, data from Amsterdam UMC, an academic hospital with a capacity of approximately 700 beds, were used. These data were not part of the development cohort. Inclusion criteria were the same as those of the development cohort. The isolates were also sampled in the period 2013 until 2022, with one isolate per patient per material per 6 months. Automated susceptibility testing was performed using the Vitek-system.

### Ethics

The Amsterdam UMC local medical ethics review committee waived the review of this study, as the medical research involving Human Subjects Act did not apply. Due to privacy restrictions, the data and scripts used for this study are not publicly available but are available upon reasonable request.

## Results

### Cohort description

The three datasets comprised a total of 178,044 isolates, tested between 2013 and 2022: 141,576 isolates of *E. coli*, 33,088 of *K. pneumoniae*, and 3,380 of *P. mirabilis* for which ESBL-confirmation testing data were available. Table 1a shows the details for this model development cohort. Prevalence of ESBL-production among the selected isolates was 84.1% for *E. coli*, 82.4% for *K. pneumoniae* and 31.6% for *P. mirabilis*. Most isolates in all subsets originated from urine samples. Around 85% of the isolates were subjected to Vitek for susceptibility testing. The cohort used for external validation (Table 1b) consisted of 5,996 isolates and had a species-distribution similar to the development cohort: 4,291 isolates of *E. coli*, 1,639 of *K. pneumoniae*, and 69 of *P. mirabilis*. Vitek was the used AST-system in this laboratory.

**TABLE 1:** Overview of datasets used in this study: a) Dataset for model development. b) Dataset for external validation.

Derivation cohort	*E. coli*	*K. pneumoniae*	*P. mirabilis*
Isolates (*n*)	141,576	33,088	3,380
ESBL-positive (%)	84.1	82.4	31.6
Unique patients (*n*)	96,270	21,821	2,765
Unique samples (*n*)	141,576	33,088	3,380
**Material (n)**			
Urine	83,020	18,332	2,164
Blood/CSF	4,837	1,189	49
Other*	53,719	13,567	1,167
**AST-system (n)**			
Vitek	120,354	27,363	2,918
Phoenix	21,222	5,725	462
**Validation cohort**	***E. coli***	***K. pneumoniae***	***P. mirabilis***
Isolates (*n*)	4,291	1,639	69
ESBL-positive (%)	92.9	95.1	62.3
Unique patients (*n*)	2,158	670	40
Unique samples (*n*)	4,038	1,590	68
**Material (n)**			
Urine	1,427	565	27
Blood/CSF	217	123	1
Other*	2,647	951	41
**SAT-system (n)**			
Vitek	4,291	1,639	69

*Other specifically consists of: genitals, respiratory, feces, pus/wound and others.

### Performance measurement

Table 2 lists the performance of the developed models across the three species. XGBoost and Random Forest achieved AUROCs of 0.97 for *E. coli* and *K. pneumoniae*, and 0.98 for *P. mirabilis*. Logistic Regression achieved an AUROC of 0.95 for *E. coli*, 0.94 for *K. pneumoniae*, and 0.93 for *P. mirabilis*. For calibration we calculated the Brier scores (the lower the better). XGBoost and Random Forest achieved Brier scores lower than 0.05 for all species. Logistic Regression achieved slightly higher Brier scores but all between 0.05 and 0.10.

**TABLE 2:** Performance of the models.

	AUROC	Brier score	Sensitivity	Accuracy	f1-score
***E. coli***					
LR	0.95 CI = (0.950, 0.954)	0.05 CI = (0.051, 0.052)	0.90 CI = (0.894, 0.899)	0.90 CI = (0.895, 0.898)	0.94 CI = (0.935, 0.937)
RF	0.97 CI = (0.968, 0.970)	0.03 CI = (0.030, 0.031)	0.96 CI = (0.955, 0.957)	0.95 CI = (0.952, 0.953)	0.97 CI = (0.971, 0.972)
XGB	0.98 CI = (0.975, 0.977)	0.03 CI = (0.028, 0.029)	0.97 CI = (0.965, 0.967)	0.96 CI = (0.959, 0.961)	0.98 CI = (0.975, 0.976)
***K. pneumoniae***					
LR	0.94 CI = (0.937, 0.944)	0.07 CI = (0.065, 0.067)	0.86 CI = (0.859, 0.867)	0.87 CI = (0.868, 0.876)	0.92 CI = (0.915, 0.920)
RF	0.97 CI = (0.963, 0.969)	0.05 CI = (0.045, 0.049)	0.92 CI = (0.912, 0.919)	0.92 CI = (0.916, 0.922)	0.95 CI = (0.947, 0.951)
XGB	0.97 CI = (0.968, 0.972)	0.04 CI = (0.042, 0.046)	0.93 CI = (0.929, 0.933)	0.93 CI = (0.928, 0.933)	0.96 CI = (0.955, 0.958)
***P. mirabilis***					
LR	0.93 CI = (0.917, 0.944)	0.08 CI = (0.076, 0.090)	0.60 CI = (0.577, 0.619)	0.86 CI = (0.853, 0.870)	0.73 CI = (0.713, 0.749)
RF	0.98 CI = (0.979, 0.987)	0.04 CI = (0.033, 0.040)	0.79 CI = (0.771, 0.811)	0.93 CI = (0.920, 0.937)	0.87 CI = (0.858, 0.890)
XGB	0.98 CI = (0.981, 0.987)	0.03 CI = (0.030, 0.039)	0.89 CI = (0.878, 0.903)	0.96 CI = (0.950, 0.960)	0.93 CI = (0.918, 0.934)

Each model was tested through Stratified 10-fold cross-validation. Scores are an average of the performances of the ten folds. A 95% confidence interval (CI) was used. See Supplementary Table 3 for definitions of the metrics. AUROC, Area Under Receiver Operating Characteristics; LR, Logistic Regression; RF, Random Forest; XGB, XGBoost.

We calculated the additional metrics (indicating the models’ discriminative abilities) with the given decision threshold (0.8). XGBoost achieved a sensitivity of 0.97, 0.93, and 0.89 for, respectively, *E. coli*, *K. pneumonia*, and *P. mirabilis*. Accuracy scores achieved by XGBoost were 0.96, 0.93, and 0.96 for, respectively, *E. coli*, *K. pneumonia*, and *P. mirabilis*. The f1-scores were also high: 0.98, 0.96, and 0.93 for, respectively, *E. coli*, *K. pneumonia*, and *P. mirabilis*. Random Forest had comparable results.

The sensitivity scores achieved by Random Forest were similar with the exception of 0.79 for *P. mirabilis*. Logistic Regression had notably lower scores. The narrower confidence intervals indicate that the results of the cross-validation splits were more stable for *E. coli* and *K. pneumoniae* compared to *P. mirabilis*. Specificity and Negative Predictive Value are added in Supplementary Table 3.

**FIGURE 1:**
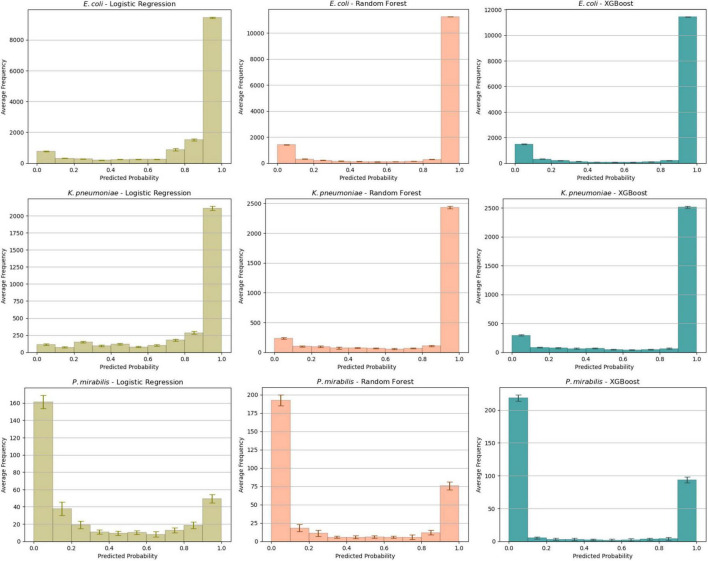
Average probability distributions in the test set for each combination of species and algorithm. The average is calculated across all ten folds with the error bars indicating the standard deviation of the mean.

We analyzed the predicted probability of the development cohort for the different models (Figure 1). This showed comparable profiles for *E. coli* and *K. pneumoniae*. As expected, most isolates had a high probability of ESBL-production. The probability distribution of *P. mirabilis* had a different profile: most isolates had a low probability. This aligns with our expectations as the ESBL-prevalence was much lower in this subset.

**FIGURE 2:**
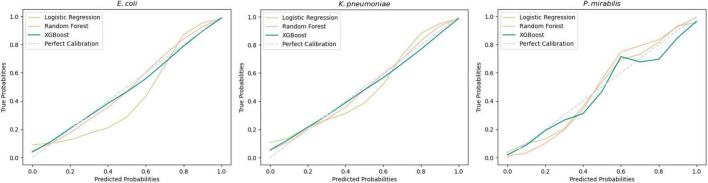
Average calibration curves across all 10-fold for each developed model. A perfect calibration represents a theoretical shape of the model without error.

Subsequent model evaluation was done by analysis of the calibration curves during cross-validation (Figure 2). For *E. coli* and *K. pneumoniae*, both the XGBoost and Random Forest models showed good overall calibration, although Random Forest tended to underestimate probabilities at lower predicted values. Logistic Regression was slightly less well-calibrated but remained close to the ideal line. For *P. mirabilis*, Logistic Regression aligned more closely with the perfect calibration line, whereas XGBoost and Random Forest overestimated probabilities in the mid-range. Despite these deviations, both models achieved higher overall performance scores (Table 1) Further details are provided in the Supplementary Figure 1.

Due to the low occurrence of Phoenix data in our dataset we conducted a subset validation of a Phoenix dataset. These results showed that there was no impact from the low occurrence of Phoenix data (Supplementary Tables 4, 5).

### Feature importance

**FIGURE 3:**
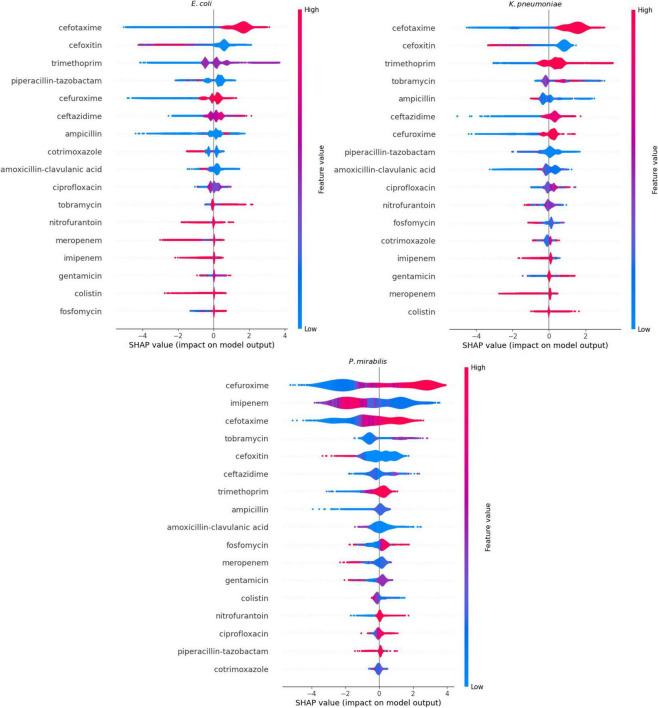
Feature importance calculated through SHapley Additive exPlanations (SHAP)-values of the XGBoost models. When the impact on the model output is larger than 0 on the X-axis, the feature value contributes to the prediction of a positive Extended Spectrum β-Lactam (ESBL)-confirmation. When the impact on the model output is lower than 0, the contribution is toward a negative outcome of ESBL-confirmation. The color represents the actual value of the feature at that particular prediction: blue represents a low MIC-value, and red a high MIC-value.

We performed the feature importance analysis only on XGBoost (Figure 3). The SHAP-values showed that the models for *E. coli* and *K. pneumoniae* had very similar ranking, with the same antibiotics in the three most important features and showing nearly identical distributions. Cefotaxime was the most important feature in both models, with a high MIC-value indicating a high predictive value for ESBL. In contrast, cefoxitin (second most important feature) showed a reverse pattern, with high values indicating a low risk of ESBL. Trimethoprim, piperacillin-tazobactam and cefuroxime completed the five most important features in both models. For the *P. mirabilis*-model, SHAP-values showed cefuroxime as the most important feature, followed by imipenem, cefotaxime, tobramycin, and cefoxitin.

### External validation

We performed the external validation on the XGBoost models being the best performing model type. Table 3 shows a performance overview of the models on the external dataset. For *E. coli* only 2.33% of the isolates were mislabeled, for *K. pneumoniae* 3.42% and for *P. mirabilis* 5.80%. The *E. coli* and *P. mirabilis*-models both achieved an AUROC of 0.93, followed by the *K. pneumoniae*-model with an AUROC of 0.89. These results show that the developed model performs well when implemented in another setting. Of note, performance outcomes in this Vitek-only subcohort were comparable to those observed in the Phoenix-only subcohort, indicating that the model performs consistently across both platforms.

**TABLE 3:** Results of predictive performance of XGBoost during the external validation.

	AUROC	Brier score	Sensitivity	Accuracy	f1-score
**XGBoost validation**					
*E. coli*	0.93	0.02	0.99	0.98	0.99
*K. pneumoniae*	0.89	0.03	0.97	0.97	0.98
*P. mirabilis*	0.93	0.06	0.98	0.94	0.95

AUROC, Area Under Receiver Operating Characteristics. See Supplementary Table 2 for definitions of the metrics.

## Discussion

We developed different ML-models that predict ESBL-production solely based on MIC-values obtained from routine antimicrobial susceptibility testing. Comparing these models, XGBoost achieved high performance which shows that it is able to instantly confirm ESBL-production with good predictive accuracy, representing a significant advancement in antimicrobial resistance detection.

The most contributing antibiotics aligned with the expectations of domain experts. High MIC-values for cefotaxime led the models to predict ESBL, in line with established confirmation guidelines. The models tended toward ESBL-positivity in case of low MIC-values for cefoxitin, which aligns with the fact that ESBL-producing micro-organisms are susceptible to cefoxitin while AmpC-producers are not. MIC-values for cefoxitin therefore also provide valuable information while this antibiotic is currently not included in the screening criteria for the ESBL-confirmation test. Cefuroxime was the most contributing antibiotic in the *P. mirabilis*-model, which is different from the other models. Plasmid-mediated AmpC rarely occurs in *P. mirabilis* (Reuland et al., 2015; Shaaban et al., 2022), which is possibly the reason that the third-generation cephalosporins have less contribution to the predictions and instead the presence or absence of second-generation cephalosporin resistance becomes the most contributing factor. The analysis of feature importances revealed that, in addition to β-lactam antibiotics, MICs of several non-β-lactam antibiotics, such as trimethoprim and ciprofloxacin, also contributed to the prediction models. This reflects the common co-resistance observed in ESBL-positive isolates.

**FIGURE 4:**
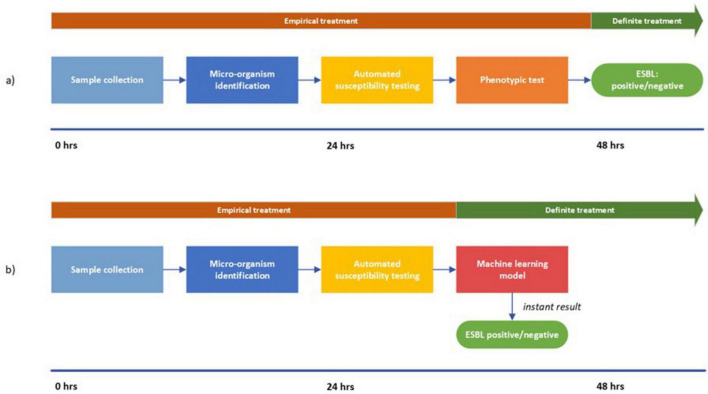
Visualization of the workflow from sample collection to ESBL-detection. **(a)** represents the current situation; **(b)** represents the proposed approach. In **(b)** the timeline is shortened due to the instant results from the machine learning model. Consequently, the switch from empirical treatment to definite treatment can be made after 24 h.

In the current situation, ESBL-production is confirmed approximately 2 days after the sample was taken from the patient (Figure 4a). Awaiting ESBL-confirmation, broad-spectrum empirical treatment is initiated before the ESBL-confirmation test results are available so as not to delay treatment. This is suboptimal as there is still the possibility of a negative test result: almost 20% for *E. coli* and *K. pneumoniae* and more than 50% for *P. mirabilis* in our study turned out not to be ESBL-producing. In recent years, several biochemical and immunological tests have been reported that can accurately detect ESBL-production and have a short time-to-results (Boattini et al., 2022; Demord et al., 2021). These tests however, require additional handling of the isolate and an incubation step. Deployment of our models could provide results without additional handling and would improve the current workflow (Figure 4b): shortening the timeline to ESBL-detection through instant results from the ML-model. Consequently, de-escalating from empirical treatment to definite treatment is possible on the second day instead of the third day, thereby preventing unnecessary use of reserve antibiotics.

The following points are important when interpreting our results. First, ML-models are based on a decision threshold that is determined on their context (permitting more false positives or more false negatives). Creating a strict model (high threshold) prevents unnecessary treatment and isolation by reducing false positives. Another approach could be to have a low decision threshold, in order to reduce false negatives as much as possible. We chose to reduce the false positives as much as possible since unnecessary use of reserve antibiotics drives increase of resistance rates. The second point is that we did not perform any hyperparameter tuning as we deemed the performance scores sufficient with the default settings of the models. Thirdly, despite the class imbalance in the datasets, we did not add class weights as the models did not show a tendency to favor the majority class. The fourth point concerns model drift. Due to real-world changes, the data that the model was trained on might no longer be representative of the current resistance levels. Retraining of the model will be required after a certain period of time.

Our approach contains several notable strengths worth highlighting. First, the traditional test requires a predefined selection criterion to limit the number of executed confirmation tests, whereas a ML-model does not require such limitations. Our ML-approach could establish a safeguard for isolates that are ESBL-positive but fall outside the predefined test criteria. Furthermore, some AST-systems have a built-in data-driven approach to pre-screen for ESBL (i.e., Advanced Expert System) that uses a proprietary database which is not open-source. Our data-driven approach, however, is directly based on the golden standard for phenotypic ESBL-confirmation which is in line with the recommendations by the European Committee on Antimicrobial Susceptibility Testing (EUCAST) and the Clinical and Laboratory Standards Institute (CLSI) (Imkamp et al., 2022; Poulou et al., 2014). Additionally, the two most commonly used AST-systems, Vitek and Phoenix, were included in the development cohort. Besides, we included the MIC-values of all antibiotics that can be found on the susceptibility cards of these AST-systems. This facilitates opportunities for new insights on association of MIC-values of specific antibiotics and ESBL-production. Lastly, the cohort represents national-level data over multiple years, making the results generalizable for Netherlands.

There are also limitations to this study. Despite the models’ discriminatory power, some cases were still misclassified. Developing an implementation strategy where both the traditional test and the ML-model are used, could reduce the missed cases. For example, one could implement the policy that every negative predicted case (the minority of cases) is still subjected to a traditional test. Furthermore, it is known that phenotypic confirmation testing is not perfectly accurate (Drieux et al., 2008). The specific type of phenotypical test used for each isolate, combination disk diffusion test or gradient test, remained unknown. Thus, we are not able to verify the universality of the models. Moreover, the ESBL-prevalence in the *P. mirabilis*-dataset is much lower than in the other datasets. This could be an explanation for the less stable models developed on this subset. An additional study to validate this with a larger cohort would be interesting. Fourth, as Netherlands has a low ESBL-prevalence, the validity of these models might be restricted to epidemiological contexts with a comparable low prevalence. We plan to validate our approach on international data from a high ESBL-prevalence region. Next, it would be interesting to know more details on the confirmation test results, i.e., details on diffusion zones for, cefotaxime or ceftazidime. This could allow the model to make better correlations with the MIC-values. Finally, the difference between amoxicillin and amoxicillin-clavulanic acid would be an interesting added feature but the windows of the AST-systems made this impossible.

A future research suggestion would be to include isolates with an MIC-value that lies below the cut-off MIC-value for third-generation cephalosporins, i.e., that do not meet the criteria for a phenotypic confirmation test. Through the MIC-values of the other antibiotics, the model could possibly predict ESBL-production accurately, whereas they would currently be missed. In addition, it would be interesting to compare the internal flagging systems of individual AST-systems to our machine-agnostic model.

In conclusion, ML-models for prediction of ESBL-production based on routine AST-data resulted in high-performing prediction models. Implementing these models in laboratory practice could significantly shorten time-to-detection. Once, deployed, this approach could facilitate widespread screening for phenotypic ESBL-production.

## Data Availability

The data analyzed in this study is subject to the following licenses/restrictions: due to privacy restrictions, the data and scripts used for this study are not publicly available but are available upon reasonable request. Requests to access these datasets should be directed to RS, r.schade@amsterdamumc.nl.

## Members of the ISIS-AR study group

J.W.T. Cohen Stuart, Noordwest Ziekenhuisgroep, Department of Medical Microbiology, Alkmaar; D.C. Melles, Meander Medical Center, Department of Medical Microbiology, Amersfoort; K. van Dijk, Amsterdam UMC, University of Amsterdam, Department of Medical Microbiology and Infection Prevention, Amsterdam Infection and Immunity Institute, Amsterdam; A. Alzubaidy, Atalmedial, Department of Medical Microbiology, Amsterdam; M. Scholing, OLVG Lab BV, Department of Medical Microbiology, Amsterdam; S.D. Kuil, Public Health Service, Public Health Laboratory, Amsterdam; G.J. Blaauw, Gelre Hospitals, Department of Medical Microbiology and Infection prevention, Apeldoorn; ISIS-AR Projectteam: W. Altorf - van der Kuil, S.M. Bierman, S.C. de Greeff, S.R. Groenendijk, R. Hertroys, L. Kruithof, I.M. Nauta, D.W. Notermans, J. Polman, W.J. van den Reek, A.F. Schoffelen, F. Velthuis, C.C.H. Wielders, B.J. de Wit, R.E. Zoetigheid, Centre for Infectious Disease Control (CIb), National Institute for Public Health and the Environment (RIVM), Bilthoven, Netherlands; W. van den Bijllaardt, Microvida Amphia, Laboratory for Microbiology and Infection Control, Breda; E.M. Kraan, IJsselland hospital, Department of Medical Microbiology, Capelle a/d Ijssel; M.B. Haeseker, Reinier de Graaf Group, Department of Medical Microbiology, Delft; J.M. da Silva, Deventer Hospital, Department of Medical Microbiology, Deventer; E. de Jong, Slingeland Hospital, Department of Medical Microbiology, Doetinchem; B. Maraha, Albert Schweitzer Hospital, Department of Medical Microbiology, Dordrecht; A.J. van Griethuysen, Gelderse Vallei Hospital, Department of Medical Microbiology, Ede; B.B. Wintermans, Admiraal De Ruyter Hospital, Department of Medical Microbiology, Goes; M.J.C.A. van Trijp, Groene Hart Hospital, Department of Medical Microbiology and Infection Prevention, Gouda; A.E. Muller, Haaglanden MC, Department of Medical Microbiology, ‘s-Gravenhage; M. Wong, Haga Hospital, Department of Medical Microbiology, ’s-Gravenhage; A. Ott, Certe, Medical Microbiology Groningen|Drenthe, Groningen; E. Bathoorn, University of Groningen, University Medical Center, Department of Medical Microbiology, Groningen; M. Lokate, University of Groningen, University Medical Center, Department of Medical Microbiology, Groningen; J. Sinnige, Regional Public Health Laboratory Haarlem, Haarlem; D.C. Melles, St Jansdal Hospital, Department of Medical Microbiology, Harderwijk; L. Bank, St Jansdal Hospital, Department of Medical Microbiology, Harderwijk; N.H. Renders, Jeroen Bosch Hospital, Department of Medical Microbiology and Infection Control, ’s-Hertogenbosch; J.W. Dorigo-Zetsma, CBSL, Tergooi MC, Department of Medical Microbiology, Hilversum; L.J. Bakker, CBSL, Tergooi MC, Department of Medical Microbiology, Hilversum; W. Ang, Comicro, Department of Medical Microbiology, Hoorn; K. Waar, Certe, Medical Microbiology Friesland|NOP, Leeuwarden; M.T. van der Beek, Leiden University Medical Center, Department of Medical Microbiology, Leiden; M.A. Leversteijn-van Hall, Eurofins Clinical Diagnostics, Department of Medical Microbiology, Leiden-Leiderdorp; S.P. van Mens, Maastricht University Medical Centre, Department of Medical Microbiology, Infectious Diseases and Infection Prevention, Maastricht; E. Schaftenaar, St Antonius Hospital, Department of Medical Microbiology and Immunology, Nieuwegein; M.H. Nabuurs-Franssen, Canisius Wilhelmina Hospital, Department of Medical Microbiology and Infectious Diseases, Nijmegen; I. Maat, Radboud University Medical Center, Department of Medical Microbiology, Nijmegen; P.D.J. Sturm, Laurentius Hospital, Roermond; B.M.W. Diederen, Bravis Hospital, Department of Medical Microbiology, Roosendaal; L.G.M. Bode, Erasmus University Medical Center, Department of Medical Microbiology and Infectious Diseases, Rotterdam; D.S.Y. Ong, Franciscus Gasthuis and Vlietland, Department of Medical Microbiology and Infection Control, Rotterdam; M. van Rijn, Ikazia Hospital, Department of Medical Microbiology, Rotterdam; S. Dinant, Maasstad Hospital, Department of Medical Microbiology, Rotterdam; M. den Reijer, Star-SHL, Rotterdam; D.W. van Dam, Zuyderland Medical Centre, Department of Medical Microbiology and Infection Control, Sittard-Geleen; E.I.G.B. de Brauwer, Zuyderland Medical Centre, Department of Medical Microbiology and Infection Control, Sittard-Geleen; R.G. Bentvelsen, Microvida ZorgSaam, Department of Medical Microbiology, Terneuzen; A.L.M. Vlek, Diakonessenhuis, Department of Medical Microbiology and Immunology, Utrecht; M. de Graaf, Saltro Diagnostic Centre, Department of Medical Microbiology, Utrecht; A. Troelstra, University Medical Center Utrecht, Department of Medical Microbiology, Utrecht; K.B. Gast, Eurofins-PAMM, Department of Medical Microbiology, Veldhoven; M.P.A. van Meer, Rijnstate Hospital, Laboratory for Medical Microbiology and Immunology, Velp; J. de Vries, VieCuri Medical Center, Department of Medical Microbiology, Venlo; J.D. Machiels, Isala Hospital, Laboratory of Medical Microbiology and Infectious Diseases, Zwolle; Parts of this work have been presented at ESCMID Global 2024.
