# Supplementary material for: Rapid extended-spectrum beta-lactamase-confirmation by using a machine learning model directly on routine automated susceptibility testing results
Source: Front Microbiol. 2025 Apr 30;16:1582703. doi: 10.3389/fmicb.2025.1582703 (PMC12075368; doi:10.3389/fmicb.2025.1582703)
Supplement: Supplementary file 1 [file Supplementary_file_1.docx]

# Supplementary material

**Table S1. MIC-ranges for the included automated AST-systems**

|  | Vitek* (mg/L) | Phoenix* (mg/L) | Ranges to consider for the feature importance (mg/L) |
| --- | --- | --- | --- |
| ampicillin | 2-32 | 4-16 | 2-32 |
| amoxicillin-clavulanic acid | 2-32 | 2-6 | 2-32 |
| cefotaxime** | 0.25-64 | 1-32 | 0.25-64 |
| cefoxitin | 4-64 | 4-16 | 4-64 |
| ceftazidime | 0.125-64 | 1-16 | 0.125-64 |
| cefuroxime | 4-16 | 1-64 | 1-64 |
| ciprofloxacin | 0.25-4 | 0.5-2 | 0.25-4 |
| colistin | 0.5-16 | 0.5-4 | 0.5-16 |
| cotrimoxazole | 2-8 | 2-8 | 2-8 |
| fosfomycin | 16-256 | 16-256 | 16-256 |
| gentamicin | 1-16 | 2-8 | 1-16 |
| imipenem | 0.25-16 | 1-8 | 0.25-16 |
| meropenem | 0.25-16 | 1-8 | 0.25-16 |
| nitrofurantoin | 16-512 | 16-64 | 16-512 |
| piperacillin-tazobactam | 4-128 | 4-64 | 4-128 |
| tobramycin | 1-16 | 2-8 | 1-16 |
| trimethoprim | 0.5-16 | 1-8 | 0.5-16 |

* Included cards are Vitek AST-N344, -N199 and -N200, Phoenix 448505 NMIC/ID-4 and 448783 NMIC/ID-95
**one Phoenix card contained ceftriaxone instead of cefotaxime. For the isolates tested on this card, the MIC-values for ceftriaxone were processed as MIC-values for cefotaxime.

**Table S2. Definitions of the performance metrics**

| AUROC | The area under the curve of true positive rate and the false positive rate. This quantifies the model’s ability to discriminate between positive and negative cases. | - |
| --- | --- | --- |
| Brier | The mean squared difference between predicted probabilities and the actual outcome. This assesses the accuracy of probabilistic predictions - the lower the score, the better the performance. | - |
| Sensitivity | The proportion of actual positive cases that are correctly classified. This quantifies the model’s ability to distinguish positive cases from the rest. | TP / (TP + FN) |
| Accuracy | The proportion of correctly classified over all cases | (TP + TN) / (TP + TN + FP + FN) |
| Precision | The proportion of true positive predictions among all positive predictions. This indicates how reliable a positive prediction is (Positive Predictive Value). | TP / (TP + FP) |
| F1-score | The harmonic mean of precision and sensitivity. | (2 x Precision x Sensitivity) / (Precision + Sensitivity) |
| Specificity | The proportion of actual negative cases that are correctly classified. This quantifies the model’s ability to distinguish negative cases from the rest. | TN / (TN + FP) |
| Negative Predictive Value | The proportion of negative predictions that are actually negative. This indicates how reliable a negative prediction is. | TN / (TN + FN) |

TP = True Positives; TN = True Negatives; FN = False Negatives; FP = False Positives

**Table S3. Additional performance metrics for the developed models**

| ***E. coli*** | | |
| --- | --- | --- |
|  | **Specificity** | **Negative Predictive Value** |
| **LR** | 0.89  CI = (0.888, 0.897) | 0.63  CI = (0.619, 0.640) |
| **RF** | 0.93  CI = (0.928, 0.935) | 0.80  CI = (0.799, 0.807) |
| **XGB** | 0.93  CI = (0.926, 0.933) | 0.84  CI = (0.833, 0.840) |
| ***K. pneumoniae*** | | |
|  | **Specificity** | **Negative Predictive Value** |
| **LR** | 0.92  CI = (0.907, 0.923) | 0.58  CI = (0.573, 0.593) |
| **RF** | 0.93  CI = (0.926, 0.941) | 0.70  CI = (0.695, 0.712) |
| **XGB** | 0.93  CI = (0.919, 0.936) | 0.74  CI = (0.730, 0.748) |
| ***P. mirabilis*** | | |
|  | **Specificity** | **Negative Predictive Value** |
| **LR** | 0.98  CI = (0.976, 0.990) | 0.84  CI = (0.831, 0.853) |
| **RF** | 0.99  CI = (0.989, 0.995) | 0.92  CI = (0.905, 0.925) |
| **XGB** | 0.98  CI = (0.978, 0.988) | 0.95  CI = (0.939, 0.953) |


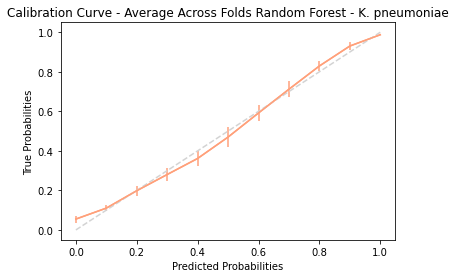

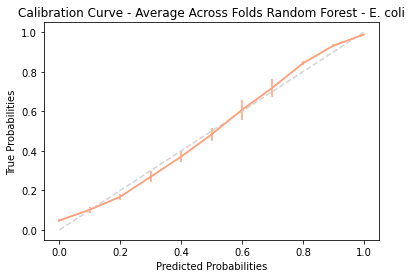

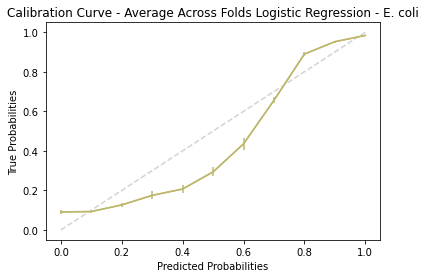

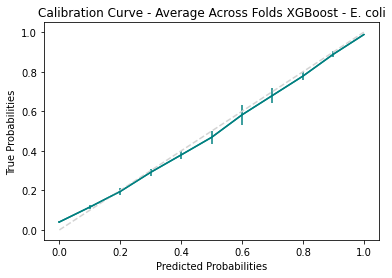

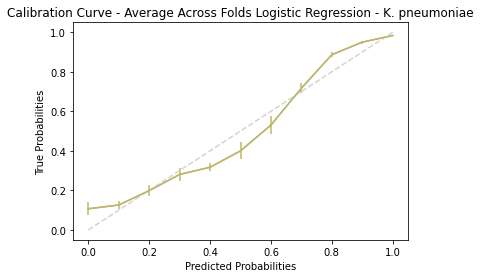

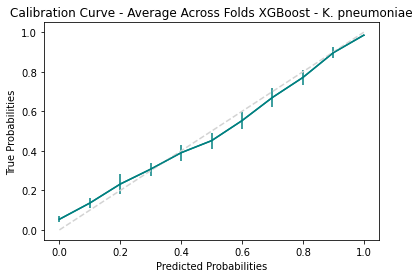

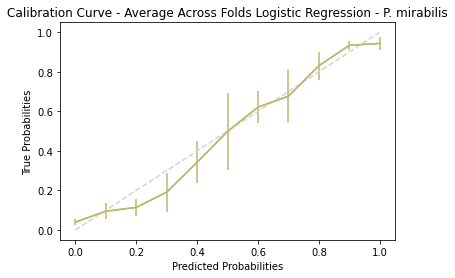

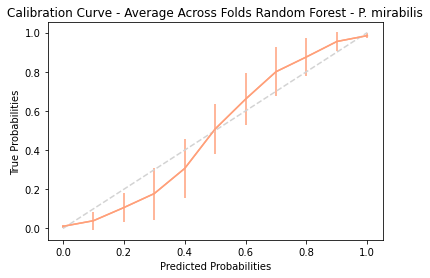

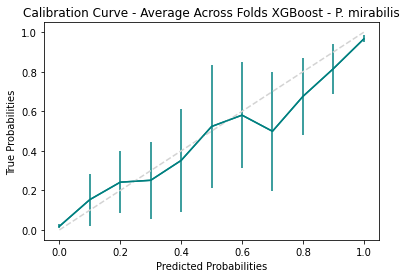


**Figure S1. Averaged calibration curves with error bars indicating the standard deviation of the mean across folds.**

**Subset validation AST system Phoenix**

We created subsets of Phoenix data using sample sizes equivalent to that of the external validation. Table S4 shows an overview of the data used for this validation. Table S5 shows the performance of the model on the Phoenix validation dataset.

**Table S4: Overview of datasets used in the Phoenix validation**

|  | *E. coli* | *K. pneumoniae* | | *P. mirabilis* |
| --- | --- | --- | --- | --- |
| Isolates (n) | 4,000 | 1,500 | 70 | |
| ESBL-positive (%) | 85.3 | 86.2 | 30.0 | |
| Unique patients (n)  Unique samples (n) | 3,605  4,000 | 1,258  1,500 | 67  70 | |
| Material (n, prev. %)  Urine  Blood/CSF  Other* | 2,220 (82.5)  117 (86.3)  1,683 (89.0) | 772 (82.4)  69 (92.8)  659 (90.0) | 47 (25.5)  3 (33.3)  20 (40.0) | |
| AST-system (n, prev. %)  Phoenix | 4,000 (85.3) | 1,500 (86.2) | 70 (30.0) | |

*Other specifically consists of: genital, respiratory, faeces, pus/wound and others.

**Table S5:** **Results of predictive performance of XGBoost during the Phoenix validation**

| **XGBoost validation** | | | | | |
| --- | --- | --- | --- | --- | --- |
|  | **AUROC** | **Brier score** | **sensitivity** | **accuracy** | **f1-score** |
| ***E. coli*** | 0.88 | 0.05 | 0.98 | 0.95 | 0.97 |
| ***K. pneumoniae*** | 0.91 | 0.05 | 0.96 | 0.95 | 0.97 |
| ***P. mirabilis*** | 0.96 | 0.04 | 0.95 | 0.96 | 0.93 |

**See Table S2 for definitions of the metrics. AUROC = Area Under Receiver Operating Characteristics.**
